# Exploring the impact of the recombinant *Escherichia coli* strain on defensins antimicrobial activity: BL21 versus Origami strain

**DOI:** 10.1186/s12934-022-01803-7

**Published:** 2022-05-09

**Authors:** Adrià López-Cano, Marc Martínez-Miguel, Judith Guasch, Imma Ratera, Anna Arís, Elena Garcia-Fruitós

**Affiliations:** 1 Department of Ruminant Production, Institut de Recerca i Tecnologia Agroalimentàries (IRTA), 08140 Caldes de Montbui, Spain; 2 Department of Molecular Nanoscience and Organic Materials, Institut de Ciència de Materials de Barcelona (ICMAB-CSIC), Campus UAB, 08193 Bellaterra, Spain; 3 Networking Research Center On Bioengineering, Biomaterials and Nanomedicine (CIBER-BBN), Madrid, Spain; 4 Dynamic Biomimetics for Cancer Immunotherapy, Max Planck Partner Group, ICMAB-CSIC, Campus UAB, 08193 Bellaterra, Spain

**Keywords:** Host defense peptides, *Escherichia coli*, Strain, Recombinant protein

## Abstract

The growing emergence of microorganisms resistant to antibiotics has prompted the development of alternative antimicrobial therapies. Among them, the antimicrobial peptides produced by innate immunity, which are also known as host defense peptides (HDPs), hold great potential. They have been shown to exert activity against both Gram-positive and Gram-negative bacteria, including those resistant to antibiotics. These HDPs are classified into three categories: defensins, cathelicidins, and histatins. Traditionally, HDPs have been chemically synthesized, but this strategy often limits their application due to the high associated production costs. Alternatively, some HDPs have been recombinantly produced, but little is known about the impact of the bacterial strain in the recombinant product. This work aimed to assess the influence of the *Escherichia coli* strain used as cell factory to determine the activity and stability of recombinant defensins, which have 3 disulfide bonds. For that, an α-defensin [human α-defensin 5 (HD5)] and a β-defensin [bovine lingual antimicrobial peptide (LAP)] were produced in two recombinant backgrounds. The first one was an *E. coli* BL21 strain, which has a reducing cytoplasm, whereas the second was an *E. coli* Origami B, that is a strain with a more oxidizing cytoplasm. The results showed that both HD5 and LAP, fused to Green Fluorescent Protein (GFP), were successfully produced in both BL21 and Origami B strains. However, differences were observed in the HDP production yield and bactericidal activity, especially for the HD5-based protein. The HD5 protein fused to GFP was not only produced at higher yields in the *E. coli* BL21 strain, but it also showed a higher quality and stability than that produced in the Origami B strain. Hence, this data showed that the strain had a clear impact on both HDPs quantity and quality.

**Supplementary Information:**

The online version contains supplementary material available at 10.1186/s12934-022-01803-7.

## Background

Infections caused by antimicrobial resistant (AMR) bacteria are continuously growing, whereas available drugs for their treatment are limited and, in some cases, nonexistent [1, 2]. The current situation has led the World Health Organization (WHO) to declare AMR as one of the top 10 global public health threats facing humanity [3]. To tackle this global challenge affecting both human and animal health, research efforts are directed to the generation of alternative antimicrobial therapies including phage therapy [4], lysins [5], probiotics [6], antibodies [7], and antimicrobial proteins [8]. Among them, host defense peptides (HDPs) outstand for their broad-spectrum bactericidal activity [9, 10]. HDPs are short, cationic peptides, which are naturally produced by the innate immunity of organisms of all life forms, being key molecules for the prevention and overcoming infections [11–13]. Besides, their fast and multiple mechanisms of action hamper the development of resistances [14–17].

The different HDPs have been classified into three groups: defensins, cathelicidins, and histatins [18, 19]. Defensins are one of the most remarkable groups, widely distributed in animals and plants. Whereas invertebrate and plant defensins contain a common structure comprising an α-helix linked to a β-sheet by two disulfide bridges (CSαβ-motif) [20], mammalian defensins are characterized by an antiparallel β-sheet structure, stabilized by three disulfide bonds [13]. In addition, mammalian defensins are divided into α- and β-defensins, which mainly differ in length, location, and connectivity of their three pairs of intramolecular disulfide bonds, as well as in their unique consensus sequences [21]. The α-defensins, which are mainly produced by neutrophils and Paneth cells in the small intestine, are 29–35 residues long, containing six cysteines which are linked as follows: C1–C6, C2–C4, and C3–C5 [22–26]. In contrast, β-defensins produced by epithelial cells are 38–42 residues long with C1–C5, C2–C4, C3–C6 pairs forming disulfide bonds [24–27]. The conserved cysteines of defensins have led to the conclusion that correct disulfide bond formation could be critical for biological activity, structuration, and stability of these peptides [28].

Most studies done with defensins have used synthetic forms of these peptides. However, some of them have also been recombinantly produced [29–32]. Unlike chemical synthesis, the recombinant production of peptides is an efficient and fully scalable process with no limits in peptide length [33–36]. Generally, when using the recombinant production strategy, defensins (and in general HDPs) are fused to carrier proteins to avoid proteolysis [37] and minimize the toxicity of these short peptides [38–40]. Some examples of fusion carriers are thioredoxin, glutathione S-transferase (GST), small ubiquitin-related modifier (SUMO) and Green Fluorescent Protein (GFP). Although different production strategies have been explored to optimize defensins production, little is known about the disulfide bond formation of HDPs under recombinant conditions. This is particularly relevant in bacterial hosts and more specifically in *Escherichia coli* (*E. coli*) because it has a reducing cytoplasmic environment maintained by the glutaredoxin and thioredoxin pathways, that hampers disulfide bond formation [41, 42]. Commercial *E. coli* strains such as Origami (Novagen), in which the thioredoxin reductase (*trxB*) and glutathione reductase (*gor*) genes are deleted have been used to produce defensins in a more oxidizing environment. For example, Wang and coworkers have compared the production of human α-defensin 6 (HD6) in *E. coli* BL21 and Origami strains, determining that higher production yields are reached when using Origami [43]. Other authors have proven that defensins produced in *E. coli* Origami are active against different pathogenic strains [44, 45]. However, the comparison of the quality (activity) of defensins produced in these two strains has not yet been evaluated. Thus, in this study, we have determined the production yields and activity of an α-defensin and a β-defensin recombinantly produced in both oxidizing and reducing *E. coli* cytoplasm. For that, we have used both the soluble form and aggregated protein forming inclusion bodies (IBs) of the human α-defensin 5 (HD5) and β-defensin lingual antimicrobial peptide (LAP). IBs are mechanically stable protein-based nanoparticles formed during recombinant protein production processes [46]. These aggregates have already been shown to be a low-cost drug delivery system for different applications, including biocatalysis [47, 48] or biomedicine, such as antimicrobial therapy [49].

## Results

Two different defensins, HD5 and LAP were selected to perform this study (Table 1). Both HDPs, which are peptides with hydrophobic regions as well as positively charged amino acids, were fused to GFP as protein carrier.

**Table 1:**
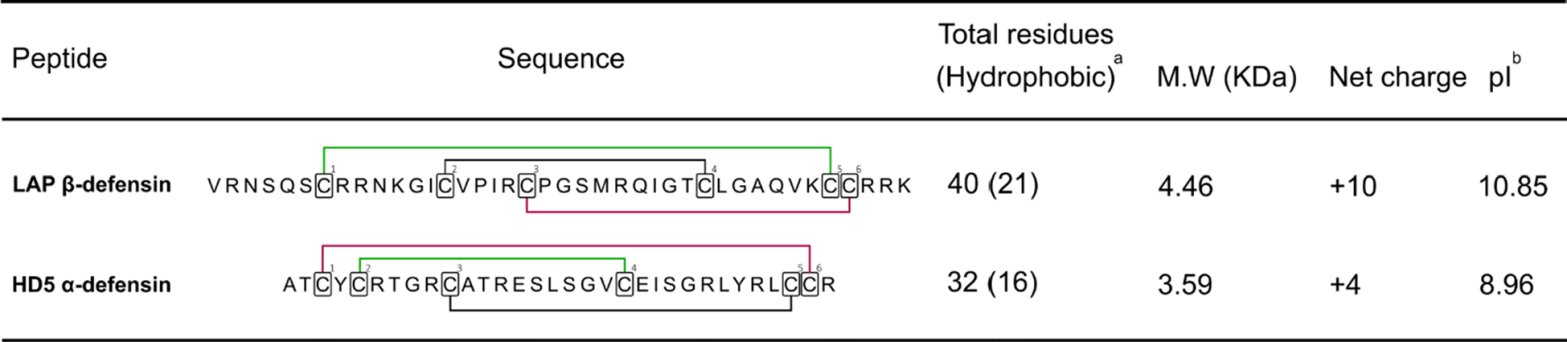
LAP (V25-K64) and HD5 (A63-R94) sequences with the disulfide cysteine pairing

The proportion of hydrophobic residues, peptide M.W, net charge and pI are also shown

*HD5* human defensin 5, *LAP* lingual antimicrobial peptide, *M.W* molecular weight, *pI* Isoelectric point

^a^The number of hydrophobic residues include amino acids with aliphatic side chains

^b^pI was theoretically calculated according to Expasy ProtParam tool

**Fig. 1:**
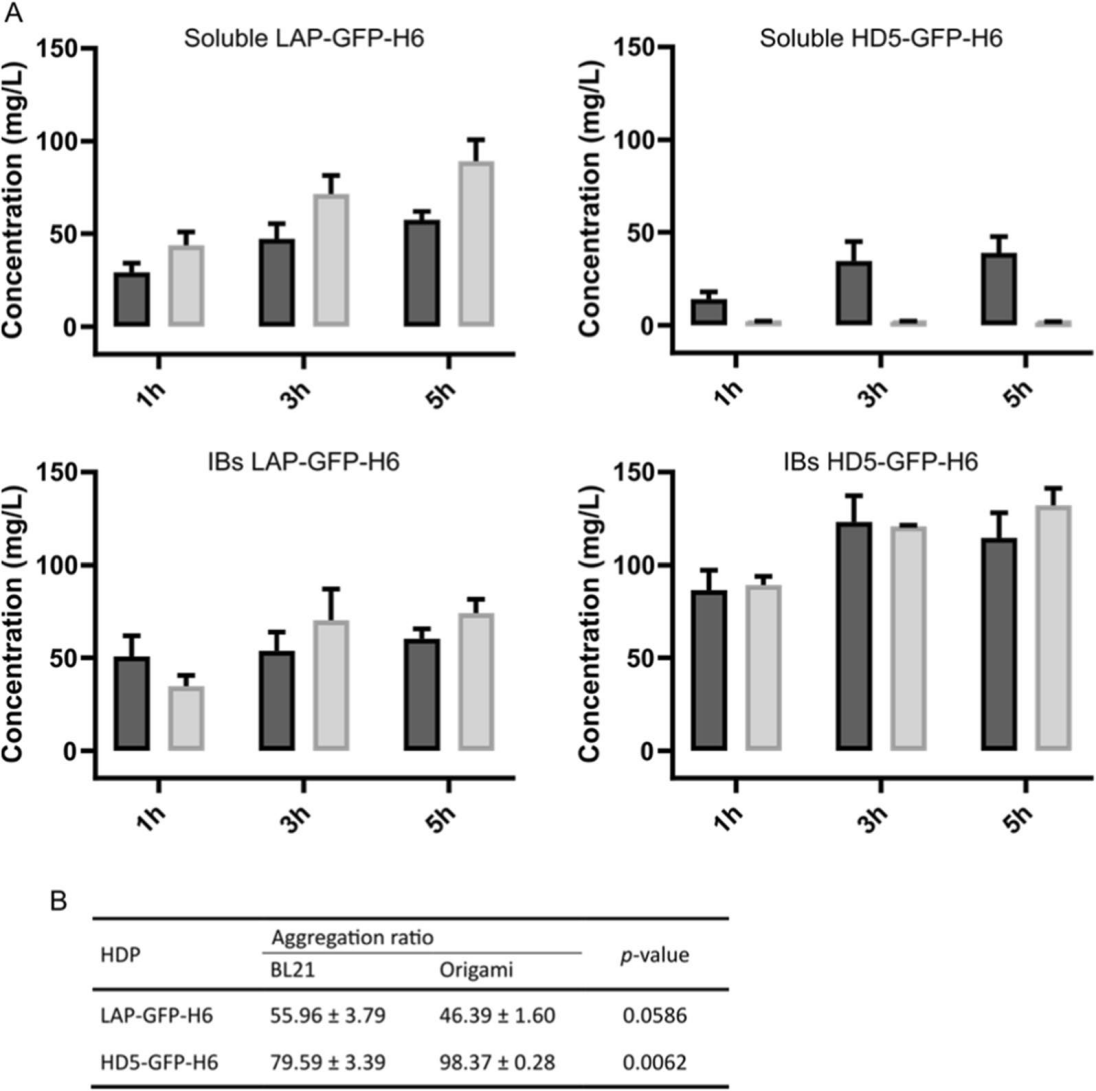
**A** Production kinetics and distribution of soluble (top) and IBs (bottom) of LAP-GFP-H6 and HD5-GFP-H6 proteins in mg/L at 1, 3, and 5 h in *E. coli* BL21 (dark grey) and Origami B (light grey) strains. The ratio of aggregation (at 3 h) for each HDP and strain is indicated in Table (**B**)

Both HD5-GFP-H6 and LAP-GFP-H6 defensins were successfully produced in *E. coli* BL21 and Origami B strains, although the production profile was different depending on the HDP and the strain used (Fig. 1). In both cases, the proteins were produced in soluble (Fig. 1 top) and insoluble (Fig. 1 bottom) forms, but the aggregation ratio was higher for HD5-GFP-H6 than LAP-GFP-H6, especially when using the Origami B strain (Fig. 1B). Soluble LAP-GFP-H6 had similar levels of production in both BL21 and Origami B strains, being in both cases time- dependent (*p* < 0.0001) (Fig. 1 top). In contrast, the production kinetics of HD5-GFP-H6 showed that the soluble form is produced at higher levels in BL21 than in the Origami B strain (Fig. 1 top; *p* = 0.040). However, the aggregated form of both LAP-GFP-H6 and HD5-GFP-H6 showed no differences between strains at the any production time (Fig. 1 bottom).

**Fig. 2:**
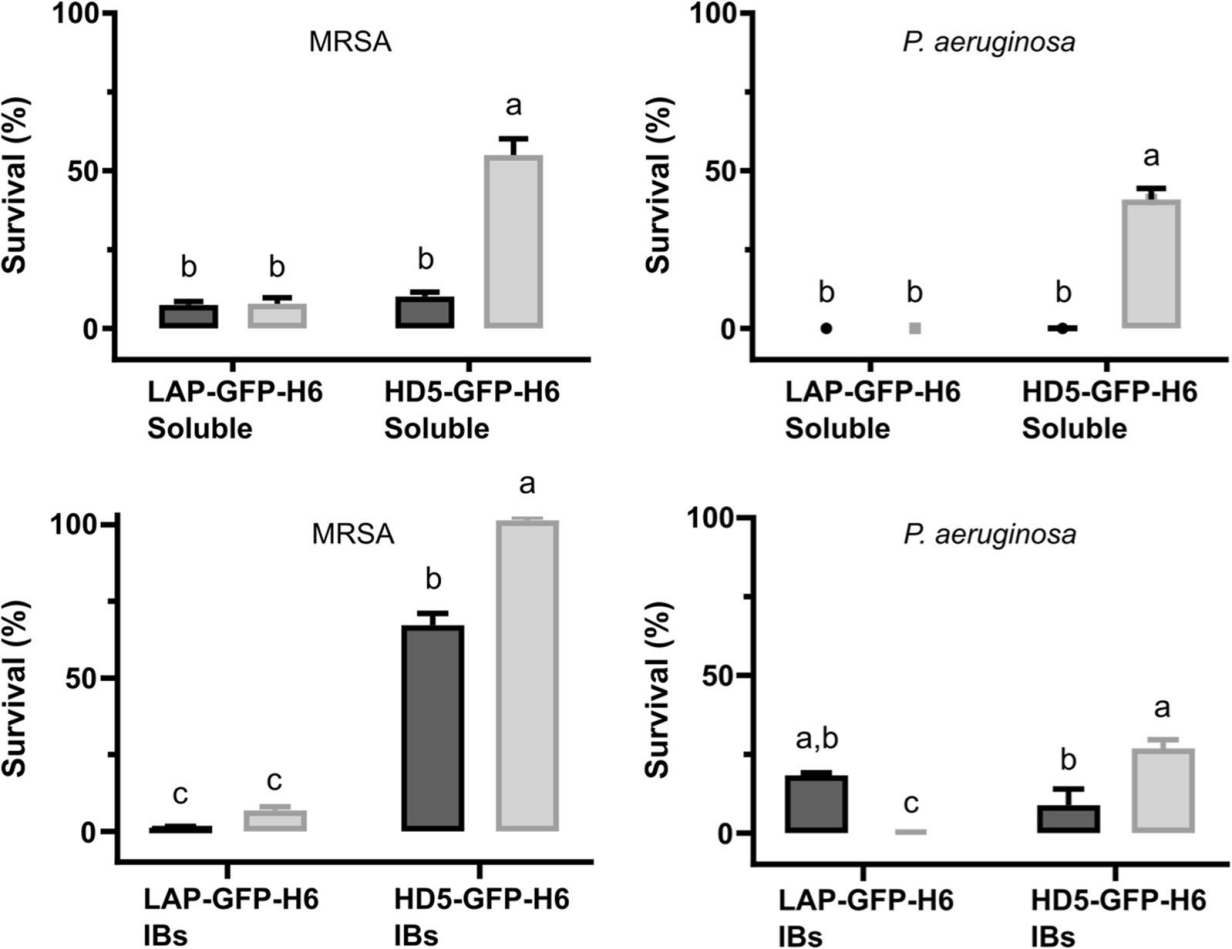
Bacterial survival of MRSA and *P. aeruginosa* in the presence of 5 μM of soluble LAP-GFP-H6 and HD5-GFP-H6 (top) and insoluble (IBs) LAP-GFP-H6 and HD5-GFP-H6 (bottom) produced in *E. coli* BL21 (dark grey) and Origami B (light grey). Different letters depict statistical differences between proteins and producer strain (MRSA soluble) *p* = 0.0024; (*P. aeruginosa* soluble) *p* < 0.0001; (MRSA IBs) *p* = 0.0108; (*P. aeruginosa* IBs) *p* = 0.094

Taking 3 h as production time, the two defensins were produced and purified in their soluble form, and the antimicrobial activity was tested against two bacterial pathogens (Fig. 2). Both defensins at 5 μM significantly reduced methicillin-resistant *Staphylococcus aureus* -MRSA- and *Pseudomonas aeruginosa* survival (Fig. 2), decreasing bacterial survival up to 99% in both organisms. By contrast, GFP alone did not show any antimicrobial activity (Additional file 1: Fig. S1). Comparing the activity of the proteins produced in a reducing environment (BL21 strain) and under more oxidizing conditions (Origami B strain), no differences were observed for LAP-GFP-H6 (Fig. 2). However, HD5-GFP-H6 produced in BL21 showed a higher bactericidal effect against both MRSA and *P. aeruginosa* (Fig. 2 top) than that produced in an Origami B strain.

To analyze the protein quality of the insoluble protein fraction of LAP-GFP-H6 and HD5-GFP-H6, bacterial IBs produced in both BL21 and Origami B strains, were purified, and their activity was tested. The results shown in Fig. 2 (bottom) proved that defensin-based IBs showed values of antimicrobial activity to levels that are comparable with the soluble fraction (Fig. 2 top). As observed with the soluble form, LAP-GFP-H6 had the same activity against MRSA regardless of the producer strain, whereas HD5-GFP-H6 IBs showed higher bactericidal activity when it was produced in a reducing environment (BL21 strain) (Fig. 2 bottom).

**Fig. 3:**
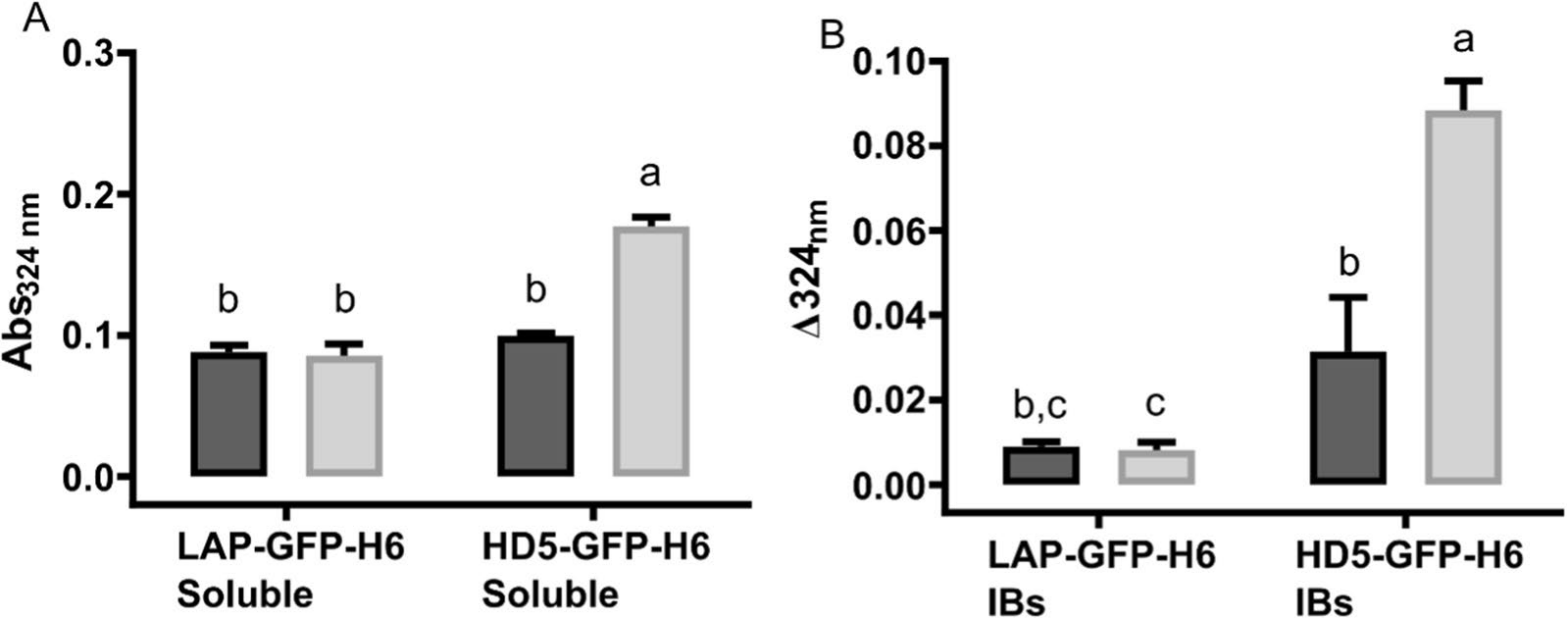
Analysis of free-cysteines in soluble (**A**) and insoluble (IBs) (**B**) LAP-GFP-H6 and HD5-GFP-H6 produced in *E. coli* BL21 (dark grey) and Origami B (light grey). Different letters depict statistical differences between proteins and strains **A** *p* = 0.0008, **B** *p* = 0.0345

The analysis of free cysteines in LAP-GFP-H6 and HD5-GFP-H6 produced in *E. coli* BL21 and Origami B strains revealed some differences (Fig. 3). Surprisingly, both soluble and insoluble (IBs) HD5-GFP-H6 had more free cysteines when using Origami as producer strain in spite of their apparent oxidizing environment than with BL21 strain (Fig. 3). In the case of LAP-GFP-H6, no differences were observed between the protein produced in both strains and forms.

**Fig. 4:**
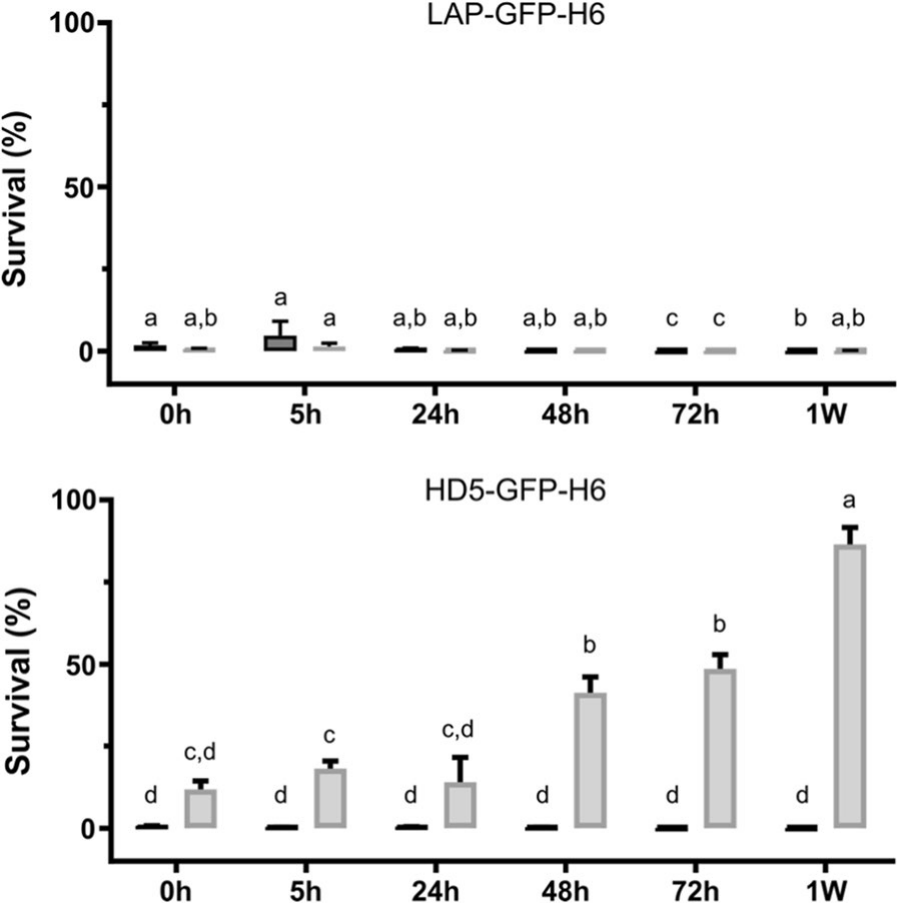
Antimicrobial activity of soluble LAP-GFP-H6 and HD5-GFP-H6 against *P. aeruginosa* at 5 μM after a 0, 5, 24, 48, 72 h and 1-week incubation at 37 °C. Dark grey bars represent the HDPs produced in *E. coli* BL21 and light grey bars represent proteins from *E. coli* Origami B strain. Different letters indicate significant statistical differences between proteins and producer strains. *p* < 0.0001. *W* week

In terms of protein stability, the analysis of the soluble LAP-GFP-H6 and HD5-GFP-H6 at 37 °C showed that the producer strain had an impact on protein stability of the α-defensin, while LAP-GFP-H6 was not affected (Fig. 4).

**Fig. 5:**
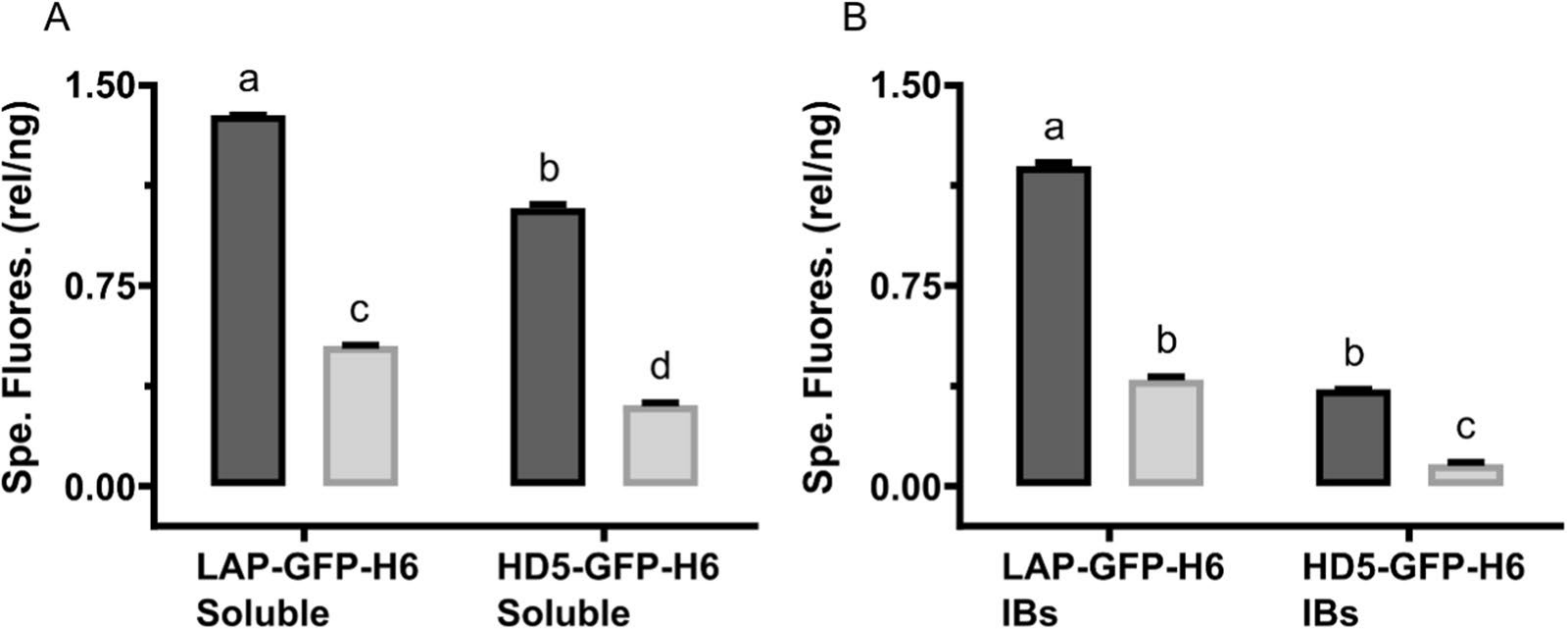
Specific GFP fluorescence (relative fluorescence units per ng of peptide) of soluble (**A**) and inclusion bodies (**B**) LAP-GFP-H6 and HD5-GFP-H6 produced in *E. coli* BL21 (dark grey) and Origami B (light grey). Different letters indicate statistical differences between proteins and strains **A** (*p* < *0.0001*) **B** (*p* < *0.0001*)

Harnessing the presence of GFP as a carrier fused to the defensins, we evaluated the potential correlation between fluorescence (Fig. 5) and antimicrobial activity of both soluble (Fig. 2 top) and insoluble (Fig. 2 bottom) of the HDPs used. However, the linear correlation values (R^2^) were in all cases lower than 0.22, which indicates a lack of interrelation between both parameters.

## Discussion

The bactericidal capacity of defensins, and in general of HDPs, has aroused the interest of the scientific community for these short peptides [13, 50]. They are part of the innate immunity and they have shown broad-spectrum activity against Gram-positive and Gram-negative bacteria, including MDR microorganisms, making them a promising alternative to antibiotic therapy [51, 52]. Structurally, HDPs have 6 cysteines that form 3 conserved disulfide bonds. In humans, among the group of α-defensins, there are two that are produced by epithelial intestinal Paneth cells (HD5 and HD6), being HD5 the most abundant enteric HDPs. These α-defensins are important as host defense against pathogens, but also maintaining intestinal homeostasis. In the group of β-defensins, LAP was one of the first described and it is expressed in tongue, mammary gland, intestine and respiratory tract [53]. Both α and β-defensins are cationic and amphiphilic peptides with a net positive charge, hydrophobicity and amphiphilic nature which allows them to interact with negatively charged bacterial cell surfaces [54]. After this interaction, HDPs have two mechanisms of action: physical disruption of bacterial cell surface and action on internal targets [54].

Different articles, in which chemically synthesized peptides have been used, reported contradictory information regarding the importance of disulfide bond formation in HDP bactericidal activity [55–60].

In terms of recombinant protein production, little is known about the impact of the producer strain in the HDPs antimicrobial activity. Classical *E. coli* strains, such as BL21 used as recombinant cell factory, have a reducing cytoplasm, while the mutant strain *E. coli* Origami has an oxidizing intracellular environment which should favor disulfide bond formation [42]. To explore the importance of cytoplasmic environment of *E. coli* strains in HDP recombinant production, we have here studied the production and activity of two HDPs (an α- and a β-defensins) in two different cytoplasmic environments. The results proved that both production yields and protein activity are not only determined by the bacterial strain used, but also by the tested peptide (Figs. 1 and 2). Whereas the β-defensin LAP fused to GFP was efficiently produced (Fig. 1A) and showed comparable activities when using both BL21 and Origami B strains (Fig. 2), HD5-GFP-H6 showed significant differences when produced in the two different bacterial backgrounds (Figs. 1 and 2). The soluble form of the HD5-GFP-H6 showed a decrease in the production yields (Fig. 1) and also a lower bactericidal activity when using an *E. coli* strain with an oxidizing environment (Origami B) (Fig. 2 top). The greater activity of the soluble α-defensin produced in the BL21 strain against both Gram-positive and Gram-negative microorganisms indicated that, contrary to expectations, this strain produced a protein with better conformational quality than that produced by Origami B strain (Fig. 2 top B). Interestingly, the soluble HD5-GFP-H6 produced in BL21(DE3) is also more active than the synthetic HDP (Additional file 1: Fig. S2).

The difference observed in activity for HD5-GFP-H6 in the two cytoplasmic environments is in agreement with the free cysteine profile observed in Fig. 3A. When comparing the HD5-GFP-H6 produced in *E. coli* BL21 and Origami B strains, the number of free cysteines is higher in the second case (Fig. 3A). This correlates with the resulting lower antimicrobial activity against the two pathogenic microorganisms tested (Fig. 2) and the diminished stability (Fig. 4). Therefore, this data supports previous works describing the importance of disulfide bonds on α-defensins stability. Tanabe et al. and Maemoto et al. reported that the disruption of disulfide bonds of HD5 and mouse α-defensin cryptdin-4, increased peptide propensity to be proteolyzed and, in consequence, the activity of these peptide variants decreased [56–58]. Thus, this shows that disulfide bonds have an important role in protein stabilization. The protein stability analysis also showed that all the HDPs with low free-cysteines produced are highly stable, keeping the bactericidal activity for at least 1 week at 37 °C (Fig. 4). This data is highly relevant in terms of applicability and storage of these bactericidal peptides.

In the same line, when the protein aggregates (IBs) were analyzed, we could observe that even though in all the cases IBs were formed (Fig. 1), the activity of HD5-GFP-H6 IBs was again significantly higher when produced in BL21 strain (Fig. 2 bottom). Moreover, both soluble (Fig. 2 top) and insoluble proteins (Fig. 2 bottom) have the same behavior in terms of protein activity. This is in line with a previous publication describing that protein conformational quality of both soluble and insoluble (IB) fractions takes place in parallel Thus, the factors affecting the conformational protein quality of the soluble form also affect the IBs [59].

Besides, this study has also proven that GFP is a good carrier protein for the production of HDPs, as other proteins such as thioredoxin, glutathione S-transferase (GST), small ubiquitin-related modifier (SUMO), or PurF fragment [60]. Indeed, GFP did not just protect the resultant HDP-based proteins from proteolytic degradation, but also simplifies protein tracking during the whole production and purification process. However, the results shown in Fig. 5 indicated that this fluorescent protein cannot be used as antimicrobial activity reporter, since the differences observed in bactericidal activity (Fig. 2) did not correlate with differences in fluorescence emission (Fig. 5).

## Conclusions

This study proved that the strain used for the production of HDP-based proteins had an impact on both the production yields and protein quality, being the *E. coli* BL21 strain an optimal background for the recombinant production of HDPs.

## Methods

### Bacterial strains and medium

*Escherichia coli* BL21 (DE3) and Origami B (DE3) (Tet^R^, Kan^R^) strains were used for heterologous protein expression. For the antibacterial assay, the strains used were *P. aeruginosa* (ATCC-10145) and methicillin-resistant *S. aureus* (MRSA. ATCC-33592). *E. coli* strains were grown in Luria–Bertani (LB) medium, whereas *P. aeruginosa* and *S. aureus* were grown in Brain–Heart Infusion (BHI) broth (Scharlau, Barcelona, Spain).

### Genetic construct design

Constructs consisting in the mature form of bovine lingual antimicrobial peptide (LAP; Uniprot entry Q28880, V25-K64) or human defensin 5 (HD5, Uniprot entry Q01523, A63-R94) were fused to green fluorescent protein (GFP) [61] using a linker sequence (SGGGSGGS) and named LAP-GFP and HD5-GFP, respectively. Each construct was C-terminally fused to a 6-histidine tag for purification and quantification purposes. LAP-GFP-H6 and HD5-GFP-H6 were codon-optimized (GeneArt^®^, Lifetechnologies, Regensburg, Germany) and cloned in pET22b (Amp^R^) (Novagene, Darmstadt, Germany) vector. pET22b vector has a T7 promoter. The plasmid with each construct (LAP-GFP-H6 HD5-GFP-H6) was transformed into competent *E. coli* BL21 (DE3) and Origami B (DE3). DE3 indicates that the host is a λDE3 lyogen, which carries a copy of T7 RNA polymerase gene under the control of *lacUV* promoter, which is induced by the presence of isopropyl-β-d-thiogalactoside (IPTG). The production of T7 RNA polymerase induce the synthesis of the protein encoded in pET22b vector with a T7 promoter.

The GFP fused to a 6 His-tag (GFP-H6) previously developed [61] was used as a control.

### Kinetics of soluble protein and inclusion body production

*Escherichia coli* BL21/pET22b cultures (0.5 L) with each antimicrobial fusion (LAP-GFP-H6, and HD5-GFP-H6) were grown overnight (O/N) in shake flasks at 37 °C and 250 rpm in LB broth with ampicillin at 100 μg/mL. *Escherichia coli* Origami B/pET22b with each antimicrobial fusion (LAP-GFP-H6 and HD5-GFP-H6) were grown at the same conditions with ampicillin, kanamycin, and tetracycline at 100, 25, and 12.5 μg/mL, respectively. The O/N were used as inoculum in fresh LB medium, starting at OD_600_ = 0.05. Recombinant protein expression was induced by 1 mM IPTG when cultures reached an OD_600_ = 0.4–0.6. Culture samples of 25 mL were withdrawn at 0, 1, 3, and 5 h post-induction, and they were collected by centrifugation at 6000×*g* for 15 min at 4 °C. Pellets were resuspended in 500 μL PBS with EDTA-free protease inhibitor (Roche) and bacteria were disrupted by sonication (2 cycles of 3 min, 0.5 s on, 0.5 s off at 10% amplitude) (Branson SFX550 Sonifier). Soluble and insoluble fractions were split by centrifugation (15,000×*g*, 15 min, 4 °C). Quantifications of LAP-GFP-H6 and HD5-GFP-H6 in both BL21 and Origami strains were obtained by western blot using a monoclonal anti-His antibody (His-probe, Santa Cruz), and their purity was evaluated by a Coomassie blue staining assay. Both outcomes were evaluated by ImageJ software to determine protein quantity and purity.

### Soluble antimicrobial protein purification

Cultures (1 L) of each fusion construct were grown and induced with IPTG, as described in the previous section. After 3 h of production, the whole culture was harvested (6000×*g*, 15 min, 4 °C). Pellets from 500 mL culture of LAP-GFP produced in both BL21 and Origami strains and HD5-GFP produced in the BL21 strain were resuspended in 30 mL of binding buffer (500 mM NaCl, 20 mM Tris, 20 mM imidazole) with EDTA-free protease inhibitor (Roche). Bacteria were sonicated (4 cycles, 5 min, 0.5 s on, 0.5 s off at 10% amplitude, Branson SFX550 Sonifier) and centrifugated (15,000×*g*, 45 min, 4 °C), collecting the supernatant, which contains soluble protein. Culture samples (1 L) of HD5-GFP produced in Origami strain was harvested (6000×*g*, 15 min, 4 °C) at 3 h post-induction, and the pellet was resuspended in 60 mL of PBS, sonicated as previously described, and centrifugated (15,000×*g*, 45 min, 4 °C). The supernatant was discarded and the pellet, containing the IBs, was washed with dH_2_O and centrifugated (15,000×*g*, 45 min, 4 °C). Then, the supernatant was discarded again, and the pellet was weighted, adding 40 mL of solubilization buffer (0.2% N-lauroylsarcosine mild detergent, 40 mM Tris) per gram of pellet. Next, the pellet was solubilized for 40 h at RT continuously stirred. Solubilized protein was recovered after centrifugation (15,000×*g*, 45 min, 4 °C), and samples were equilibrated at 500 mM NaCl and 20 mM imidazole for purification.

All soluble proteins (obtained from supernatant or solubilized IBs) were filtered using a pore diameter of 0.2 μm and purified by Immobilized Metal Affinity Chromatography (IMAC) in an ÄKTA Start (GE Healthcare) using 1 mL HiTrap chelating HP columns (GE Healthcare). Protein was loaded with binding buffer (20 mM Tris, 500 mM NaCl, 20 mM Imidazole) and eluted using a linear gradient with elution buffer (20 mM Tris, 500 mM NaCl, 500 mM Imidazole). Protein buffer exchange was done by dialysis in acetic 0.01% (v/v) O/N at 4 °C with gentle agitation. The yield of purified soluble protein was determined by NanoDrop™, and the integrity and purity of the protein were analyzed by Western blot and Coomassie.

### IB purification

As described before, at 3 h post-induction, culture was harvested (6000×*g*, 15 min, 4 °C). The supernatant was discarded and the pellet was stored at − 80 °C (minimum 16 h). Then, cells were thawed at RT, sonicated (2 cycles, 1.5 min, 0.5 s on, 0.5 s off at 10% amplitude, Branson SFX550 Sonifier), and stored at − 80 °C O/N. Next, samples were thawed, and 0.2% (v/v) Triton X-100 (Sigma Aldrich) was added, incubating for 1 h at RT and 250 rpm, sample then was frozen at − 80 °C. An extra frozen/thawed cycle was recommended. Next, a contamination control was performed, 100 μL of sample was platted on LB-agar plate and incubated at 37 °C O/N. Freeze/thaw cycles were repeated until no viable bacteria were observed in control plates. Further, IBs were incubated with 250 μL NP-40 (ThermoScientific™) for 1 h at 4 °C and 250 rpm. Afterward, 0.6 μg/mL DNase I (Roche) and 0.6 μg/mL MgSO_4_ were added, and the sample was incubated for 1 h at 37 °C and 250 rpm. Then, the IBs were collected by centrifugation (15,000×*g*, 15 min, 4 °C) and the supernatant was discarded. After, IBs were resuspended in lysis buffer (100 mM NaCl, 50 mM Tris, 1 mM EDTA, 0.5% Triton X-100), followed by a contamination control as previously described. Then, IBs were harvested (15,000×*g*, 15 min, 4 °C) and frozen − 80 °C after supernatant was removed. Finally, IBs were washed in 10 mL PBS, aliquoted, and centrifuged (15,000×*g*, 15 min, 4 °C). The supernatant was removed and the pellets, which contained purified IBs, were kept at 80 °C until use. Purity and quantity of purified IBs were assessed by Western Blot and Coomassie assay. Shortly, the samples of soluble and IBs proteins were boiled at 95 °C for 10 and 45 min, respectively, to ensure that the protein is completely denaturalized. Next, samples were resuspended with Laemmli loading buffer (100 mM Tris, 8% glycerol, 55 mM SDS, 4% β-mercaptoethanol, and 1.6% urea) and subsequent analyzed by electrophoresis (SDS-PAGE). Proteins bands were electroblotted into PVDF membranes at 1.3 A and 25 V for 10 min, followed by a blocked step at 4ºC O/N with bovine serum albumin (BSA) at 5% in TBST buffer (10 mM Tris, 150 mM NaCl, 0.05% Tween 20). Next, the membranes were incubated 2 h at RT in primary antibody (Anti-His, Sant Cruz Biotechnology) at 1:1000 dilution, followed by 3 washes in TBST buffer. Finally, membranes were incubated with secondary antibody (anti-mouse IgG-alkaline phosphatase (Sigma) at 1/20,000 dilution for 1 h at RT and three time washed. Proteins bands were revealed after alkaline phosphatase (NBT/BCIP, Thermo Scientific) substrate incubation and protein was quantified with ImageJ software.

### Antibacterial activity assay

Antimicrobial activity was determined with the Bactiter-Glo ™ Microbial Cell Viability kit (Promega). Briefly, an O/N culture of MRSA and *P. aeruginosa* was diluted 100-fold in 10 mM KPi (10 mM), aliquoted in 150 μL eppendorf, and centrifugated (6200×*g*, 15 min, 4 °C). Supernatant was removed and the bacteria pellet was resuspended in 150 μL of each treatment (acetic buffer—negative control, soluble proteins (LAP-GFP-H6 and HD5-GFP-H6) at 5 μM and IBs (LAP-GFP-H6 and HD5-GFP-H6) at 5 μM). Samples were incubated in a sterile polypropylene 96-well microtiter plate 5 h at 37 °C. Next, 100 µL of each sample were mixed with the same volume of BacTiter-Glo™ reagent on a sterile 96-well white opaque microtiter plate. Plates were incubated for 5 min and luminescence was measured in a microplate luminometer (LumiStar, Omega). The measured arbitrary luminescence values were normalized against the control (KPi treatment).

### Protein stability assay

To evaluate protein stability, soluble LAP-GFP-H6 and HD5-GFP-H6 were incubated at different timescales (0 h, 5 h, 24 h, 48 h, 72 h and 1 W) at 37 °C and then, antimicrobial activity was evaluated as previously described.

### Fluorescence measurements

Fluorescence of the GFP fused with the antimicrobial peptides was recorded in a fluorescence spectrophotometer (LumiStar, Omega). LAP-GFP-H6 and HD5-GFP-H6 in both soluble and IBs formats produced in both *E. coli* BL21 and Origami B strains were analyzed, being the samples diluted when required. They were excited at 480 nm and the emission was recorded at 510 nm. Specific fluorescence was calculated using the amount of protein in each sample.

### Sulfhydryl determination

Sulfhydryls (–SH) not-forming disulfide bonds (–S–S–) were determined according to a previously established protocol based on 4,4′-diothiodipyridine (DTDP) [62]. DTDP is a small, amphiphilic uncharged molecule, capable of quickly reacting with poorly accessible sulfhydryls. In this case, samples were diluted to a final sulfhydryl concentration ≤ 40 μM in 1 mL (calculated by multiplying protein moles by the number of SH) and mixed with 200 μL of sodium buffer (100 mM NaH_2_PO_4_, 0.2 mM EDTA, pH 6.8 adjusted with NaOH). After the addition of 50 μL of 4 mM DTDP, samples were vortexed and incubated for 5 min at RT. Next, samples were read at an absorbance of 324 nm (*A*_324_) against a water blank. For the reagent blank (*A*_324r_), 1 mL of potassium phosphate buffer was mixed with 200 μL of sodium buffer and 50 μL of DTDP reagent. For the protein blank (*A*_324p_), 50 μL of water was added instead of DTDP reagent in the sample with a 200 μL sodium buffer. For proteins in insoluble form, the A_324_ increase was monitored over time after the addition of DTDP to the sample, to achieve an increment of A_324_ over 5 min.

### Statistical analysis

All experiments were performed in triplicate and represented as the mean of non-transformed data ± non-transformed standard error of the mean (SEM). Data were previously checked for normality (JMP, SAS Institute Inc.) and *p-*values and letters correspond to the ANOVA analyses and Tukey test analyses respectively, using transforming data when required.

## Supplementary Information

**Additional file 1: Figure S1. **Bacterial survival of *P. aeruginosa* in the presence and absence of 5 μM of soluble GFP-H6. **Figure S2. **Antimicrobial activity of soluble HD5-GFP-H6 (dark grey) and synthetic HD5 (light grey) against MRSA and *P. aeruginosa*. Different letters indicate statistical differences between tested strains and proteins P = 0.0001.

## Data Availability

All data analysed during the current study are available from the corresponding author on reasonable request.

## References

[1] Blair JM, Webber MA, Baylay AJ, Ogbolu DO, Piddock LJ (2015). Molecular mechanisms of antibiotic resistance. Nat Rev Microbiol.

[2] Brown ED, Wright GD (2016). Antibacterial drug discovery in the resistance era. Nature.

[3] World Health Organization (2014). Antimicrobial resistance: global report on surveillance.

[4] Brives C, Pourraz J (2020). Phage therapy as a potential solution in the fight against AMR: obstacles and possible futures. Palgrave Commun.

[5] Fischetti VA (2008). Bacteriophage lysins as effective antibacterials. Curr Opin Microbiol.

[6] Silva DR, Sardi JdCO, de Souza Pitangui N, Roque SM, da Silva ACB, Rosalen PL (2020). Probiotics as an alternative antimicrobial therapy: current reality and future directions. J Func Foods.

[7] Zurawski DV, McLendon MK (2020). Monoclonal antibodies as an antibacterial approach against bacterial pathogens. Antibiotics.

[8] Levy O (2000). Antimicrobial proteins and peptides of blood: templates for novel antimicrobial agents. Blood.

[9] Gupta S, Bhatia G, Sharma A, Saxena S (2018). Host defense peptides: an insight into the antimicrobial world. J Oral Maxillofac Pathol.

[10] Mookherjee N, Anderson MA, Haagsman HP, Davidson DJ (2020). Antimicrobial host defence peptides: functions and clinical potential. Nat Rev Drug Discov.

[11] Haney EF, Mansour SC, Hancock RE (2017). Antimicrobial peptides: an introduction. Methods Mol Biol.

[12] Hancock RE, Lehrer R (1998). Cationic peptides: a new source of antibiotics. Trends Biotechnol.

[13] Zasloff M (2002). Antimicrobial peptides of multicellular organisms. Nature.

[14] Yeung AT, Gellatly SL, Hancock RE (2011). Multifunctional cationic host defence peptides and their clinical applications. Cell Mol Life Sci.

[15] Boparai JK, Sharma PK (2020). Mini review on antimicrobial peptides, sources, mechanism and recent applications. Protein Pept Lett.

[16] Brogden KA (2005). Antimicrobial peptides: pore formers or metabolic inhibitors in bacteria?. Nat Rev Microbiol.

[17] Hancock REW (2003). Concerns regarding resistance to self-proteins. Microbiology.

[18] De Smet K, Contreras R (2005). Human antimicrobial peptides: defensins, cathelicidins and histatins. Biotechnol Lett.

[19] Zhang LJ, Gallo RL (2016). Antimicrobial peptides. Curr Biol.

[20] Vizioli J, Salzet M (2002). Antimicrobial peptides from animals: focus on invertebrates. Trends Pharmacol Sci.

[21] Papagianni M (2003). Ribosomally synthesized peptides with antimicrobial properties: biosynthesis, structure, function, and applications. Biotechnol Adv.

[22] Selsted ME, Harwig SS (1989). Determination of the disulfide array in the human defensin HNP-2. A covalently cyclized peptide. J Biol Chem.

[23] Shi J, Ross CR, Chengappa MM, Sylte MJ, McVey DS, Blecha F (1996). Antibacterial activity of a synthetic peptide (PR-26) derived from PR-39, a proline-arginine-rich neutrophil antimicrobial peptide. Antimicrob Agents Chemother.

[24] Lehrer RI, Lichtenstein AK, Ganz T (1993). Defensins: antimicrobial and cytotoxic peptides of mammalian cells. Annu Rev Immunol.

[25] Selsted ME, Ouellette AJ (2005). Mammalian defensins in the antimicrobial immune response. Nat Immunol.

[26] Yang D, Biragyn A, Kwak LW, Oppenheim JJ (2002). Mammalian defensins in immunity: more than just microbicidal. Trends Immunol.

[27] Tang YQ, Selsted ME (1993). Characterization of the disulfide motif in BNBD-12, an antimicrobial beta-defensin peptide from bovine neutrophils. J Biol Chem.

[28] Zhang L (2017). Different dynamics and pathway of disulfide bonds reduction of two human defensins, a molecular dynamics simulation study. Proteins.

[29] Schroeder BO, Wu Z, Nuding S, Groscurth S, Marcinowski M, Beisner J (2011). Reduction of disulphide bonds unmasks potent antimicrobial activity of human β-defensin 1. Nature.

[30] Azari M, Asad S, Mehrnia MR (2020). Heterologous production of porcine derived antimicrobial peptide PR-39 in *Escherichia coli* using SUMO and intein fusion systems. Protein Expr Purif.

[31] Jin F, Xu X, Wang L, Zhang W, Gu D (2006). Expression of recombinant hybrid peptide cecropinA(1–8)-magainin2(1–12) in *Pichia pastoris*: purification and characterization. Protein Expr Purif.

[32] Corrales-Garcia LL, Possani LD, Corzo G (2011). Expression systems of human β-defensins: vectors, purification and biological activities. Amino Acids.

[33] Clement H, Flores V, Diego-Garcia E, Corrales-Garcia L, Villegas E, Corzo G (2015). A comparison between the recombinant expression and chemical synthesis of a short cysteine-rich insecticidal spider peptide. J Venom Anim Toxins Incl Trop Dis.

[34] Ongey EL, Neubauer P (2016). Lanthipeptides: chemical synthesis versus in vivo biosynthesis as tools for pharmaceutical production. Microb Cell Fact.

[35] Wegmuller S, Schmid S (2014). Recombinant peptide production in microbial cells. Curr Org Chem.

[36] Klüver E, Adermann K, Schulz A (2006). Synthesis and structure-activity relationship of beta-defensins, multi-functional peptides of the immune system. J Pept Sci.

[37] Li Y (2009). Carrier proteins for fusion expression of antimicrobial peptides in *Escherichia coli*. Biotechnol Appl Biochem.

[38] de Oliveira KBS, Leite ML, Rodrigues GR, Duque HM, da Costa RA, Cunha VA (2020). Strategies for recombinant production of antimicrobial peptides with pharmacological potential. Expert Rev Clin Pharmacol.

[39] Ingham AB, Moore RJ (2007). Recombinant production of antimicrobial peptides in heterologous microbial systems. Biotechnol Appl Biochem.

[40] Bommarius B, Jenssen H, Elliott M, Kindrachuk J, Pasupuleti M, Gieren H (2010). Cost-effective expression and purification of antimicrobial and host defense peptides in *Escherichia coli*. Peptides.

[41] Waegeman H, Soetaert W (2011). Increasing recombinant protein production in *Escherichia coli* through metabolic and genetic engineering. J Ind Microbiol Biotechnol.

[42] de Marco A (2009). Strategies for successful recombinant expression of disulfide bond-dependent proteins in *Escherichia coli*. Microb Cell Fact.

[43] Wang A, Su Y, Wang S, Shen M, Chen F, Chen M (2010). High efficiency preparation of bioactive human alpha-defensin 6 in *Escherichia coli* origami(DE3)pLysS by soluble fusion expression. Appl Microbiol Biotechnol.

[44] Su G, Xie K, Chen D, Yu B, Huang Z, Luo Y (2019). Differential expression, molecular cloning, and characterization of porcine beta defensin 114. J Anim Sci Biotechnol.

[45] Guillén-Chable F, Arenas-Sosa I, Islas-Flores I, Corzo G, Martinez-Liu C, Estrada G (2017). Antibacterial activity and phospholipid recognition of the recombinant defensin J1–1 from *Capsicum* genus. Protein Expr Purif.

[46] García-Fruitós E, González-Montalbán N, Morell M, Vera A, Ferraz RM, Arís A (2005). Aggregation as bacterial inclusion bodies does not imply inactivation of enzymes and fluorescent proteins. Microb Cell Fact.

[47] de Marco A, Ferrer-Miralles N, Garcia-Fruitós E, Mitraki A, Peternel S, Rinas U (2019). Bacterial inclusion bodies are industrially exploitable amyloids. FEMS Microbiol Rev.

[48] García-Fruitós E, Vázquez E, Díez-Gil C, Corchero JL, Seras-Franzoso J, Ratera I (2012). Bacterial inclusion bodies: making gold from waste. Trends Biotechnol.

[49] Roca-Pinilla R, López-Cano A, Saubi C, Garcia-Fruitós E, Arís A (2020). A new generation of recombinant polypeptides combines multiple protein domains for effective antimicrobial activity. Microb Cell Fact.

[50] Jenssen H, Hamill P, Hancock RE (2006). Peptide antimicrobial agents. Clin Microbiol Rev.

[51] Afacan NJ, Yeung AT, Pena OM, Hancock RE (2012). Therapeutic potential of host defense peptides in antibiotic-resistant infections. Curr Pharm Des.

[52] Wilmes M, Cammue BP, Sahl HG, Thevissen K (2011). Antibiotic activities of host defense peptides: more to it than lipid bilayer perturbation. Nat Prod Rep.

[53] Stolzenberg ED, Anderson GM, Ackermann MR, Whitlock RH, Zasloff M (1997). Epithelial antibiotic induced in states of disease. Proc Natl Acad Sci USA.

[54] Hancock RE, Sahl HG (2006). Antimicrobial and host-defense peptides as new anti-infective therapeutic strategies. Nat Biotechnol.

[55] Wanniarachchi YA, Kaczmarek P, Wan A, Nolan EM (2011). Human defensin 5 disulfide array mutants: disulfide bond deletion attenuates antibacterial activity against *Staphylococcus aureus*. Biochemistry.

[56] Maemoto A, Qu X, Rosengren KJ, Tanabe H, Henschen-Edman A, Craik DJ (2004). Functional analysis of the alpha-defensin disulfide array in mouse cryptdin-4. J Biol Chem.

[57] Tanabe H, Ayabe T, Maemoto A, Ishikawa C, Inaba Y, Sato R (2007). Denatured human alpha-defensin attenuates the bactericidal activity and the stability against enzymatic digestion. Biochem Biophys Res Commun.

[58] Zhang Y, Cougnon FB, Wanniarachchi YA, Hayden JA, Nolan EM (2013). Reduction of human defensin 5 affords a high-affinity zinc-chelating peptide. ACS Chem Biol.

[59] González-Montalbán N, García-Fruitós E, Villaverde A (2007). Recombinant protein solubility—does more mean better?. Nat Biotechnol.

[60] Silva ON, Mulder KC, Barbosa AE, Otero-Gonzalez AJ, Lopez-Abarrategui C, Rezende TM (2011). Exploring the pharmacological potential of promiscuous host-defense peptides: from natural screenings to biotechnological applications. Front Microbiol.

[61] Roca-Pinilla R, Fortuna S, Natalello A, Sánchez-Chardi A, Ami D, Arís A (2020). Exploring the use of leucine zippers for the generation of a new class of inclusion bodies for pharma and biotechnological applications. Microb Cell Fact.

[62] Riener CK, Kada G, Gruber HJ (2002). Quick measurement of protein sulfhydryls with Ellman’s reagent and with 4,4′-dithiodipyridine. Anal Bioanal Chem.

