# Supplementary material for: Exploring the impact of the recombinant Escherichia coli strain on defensins antimicrobial activity: BL21 versus Origami strain
Source: Microb Cell Fact. 2022 May 9;21:77. doi: 10.1186/s12934-022-01803-7 (PMC9082834; doi:10.1186/s12934-022-01803-7)
Supplement: Supplementary file 1 — Additional file 1: Figure S1. Bacterial survival of P. aeruginosa in the presence and absence of 5 μM of soluble GFP-H6. Figure S2. Antimicrobial activity of soluble HD5-GFP-H6 (dark grey) and synthetic HD5 (light grey) against MRSA and P. aeruginosa. Different letters indicate statistical differences between tested strains and proteins P = 0.0001. [file 12934_2022_1803_MOESM1_ESM.docx]

**Additional file 1**

**Exploring the impact of the recombinant *Escherichia coli* strain on defensins antimicrobial activity: BL21 versus Origami strain**

Adrià López-Cano^1^, Marc Martínez-Miguel^2,3^, Judith Guasch^2,3,4^, Imma Ratera^2,3^, Anna Arís^1^*and Elena Garcia-Fruitós^1^*

^1^Department of Ruminant Production, Institut de Recerca i Tecnologia Agroalimentàries (IRTA), 08140 Caldes de Montbui, Spain

^2^ Department of Molecular Nanoscience and Organic Materials, Institut de Ciència de Materials de Barcelona (ICMAB-CSIC), Campus UAB, Bellaterra 08193, Spain

^3^ Networking Research Center on Bioengineering, Biomaterials and Nanomedicine (CIBER-BBN), Spain

^4^ Dynamic Biomimetics for Cancer Immunotherapy, Max Planck Partner Group, ICMAB-CSIC, Campus UAB, Bellaterra 08193, Spain

*Corresponding author. Tel: + 34 93 467 40 40; Fax: +34 93 467 40 42; E-mail: anna.aris@irta.cat, elena.garcia@irta.cat


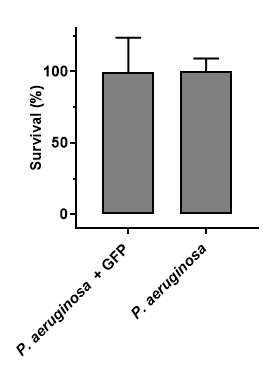


**Figure S1.** Bacterial survival of *P. aeruginosa* in the presence and absence of 5 μM of soluble GFP-H6.


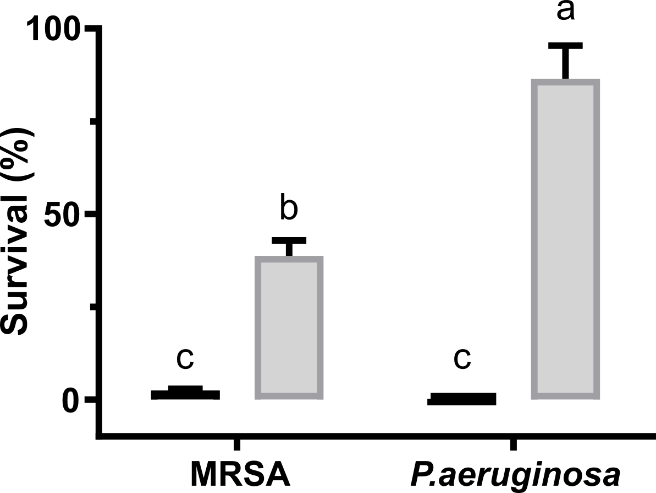


**Figure S2.** Antimicrobial activity of soluble HD5-GFP-H6 (dark grey) and synthetic HD5 (light grey) against MRSA and *P. aeruginosa*. Different letters indicate statistical differences between tested strains and proteins P=0.0001.
